# Stereotactic Radiosurgery in a Small Cell Lung Cancer Patient With Numerous Brain Metastases

**DOI:** 10.7759/cureus.28431

**Published:** 2022-08-26

**Authors:** Andrew Lian, Colton Ladbury, Arya Amini

**Affiliations:** 1 Radiation Oncology, Western University of Health Sciences, Pomona, USA; 2 Radiation Oncology, City of Hope National Medical Center, Duarte, USA

**Keywords:** small cell lung cancer, whole-brain radiation, general radiation oncology, brain met, brain stereotactic radiosurgery

## Abstract

Small cell lung cancer (SCLC) is an aggressive form of lung cancer characterized by its propensity to metastasize to the brain. When SCLC patients develop brain metastasis, the standard-of-care treatment is whole-brain radiotherapy (WBRT), with the goal of treating both macroscopic and microscopic tumors. However, WBRT is found to be associated with significant morbidity including cognitive impairment. An emerging alternative to WBRT for SCLC is stereotactic radiosurgery (SRS), supported by a recent multi-institutional series and meta-analysis. However, there is limited evidence on the use of SRS when there are greater than 15 lesions from any histology, much less SCLC, where the risk of microscopic disease is felt to be even higher. Here, we present the case of an adult female with extensive-stage SCLC who developed 23 brain metastases. Due to patient preference, these were treated with SRS to a total dose of 20 Gy in one fraction. The patient did not experience any radiation-induced toxicity, including radionecrosis, and had overall favorable intracranial control using SRS alone at the time of her death, which was due to extracranial disease progression. This case adds to the literature suggesting that SRS could be a reasonable option for patients with SCLC. It illustrates that it might be reasonable to seek to expand on who might be considered a candidate for SRS treatment, with a high number of lesions not necessarily representing imminent widespread intracranial disease progression.

## Introduction

Small cell lung cancer (SCLC) is an aggressive form of lung cancer, making up approximately 13% of all newly diagnosed lung cancers [1]. In particular, SCLC is characterized by its propensity to metastasize to the brain in approximately 40%-50% of patients throughout the course of the disease [2]. When SCLC patients develop brain metastasis, the standard-of-care treatment is whole-brain radiotherapy (WBRT) [3,4]. This treatment utilizes radiation to the entirety of the brain in hopes to kill all tumor cells present, both macroscopic and microscopic, usually requiring 10 daily treatments over the course of two weeks [5]. However, WBRT is found to be associated with significant morbidity including cognitive impairment [6]. Furthermore, with such aggressive cancer, treatment often still has a poor prognosis of 2-6 months, and therefore, the availability of more convenient and less toxic treatment options is critical [7].

Another method that has also been used for treating brain metastases from other cancer types is stereotactic radiosurgery (SRS). The key advantage of SRS over WBRT is its ability to limit radiation dose to the unaffected brain, ultimately limiting side effects and facilitating more rapid treatment delivery [8]. Historically, SRS has not been recommended for brain metastases from SCLC due to the perceived high risk of microscopic disease elsewhere in the brain that could prove problematic and potentially life-limiting if left untreated. Recently, data emerged from the First-line Radiosurgery for Small-Cell Lung Cancer Brain Metastases (FIRE-SCLC) study demonstrating the feasibility of using SRS for SCLC brain metastasis. The FIRE-SCLC study was a multi-institutional retrospective study of patients with SCLC brain metastases who were treated with either SRS or WBRT [9]. Patients treated with SRS had a median of 2.5 metastases with an interquartile range of 1-6. While, as expected, intracranial control was inferior in patients treated with SRS, overall survival (OS) was actually improved in patients receiving SRS. In subsequent meta-analyses by Gaebe et al. [10] and Viani et al. [11], these results were replicated, with SRS being associated with an improvement in OS, thereby establishing SRS as a viable treatment option. Importantly, these studies are limited by the inclusion of retrospective studies and associated selection bias but still support consideration of SRS in future studies.

An ongoing debate in radiation oncology is whether there is a limit to the number of lesions that can be treated with SRS, which is even more controversial in SCLC, where a large number of brain metastases would be felt to indicate disseminated intracranial disease. There is randomized data for the treatment of up to 15 lesions in non-SCLC histologies [12]. Data from treating more than 15 lesions is limited and in the case of SCLC largely nonexistent. Herein, we present the case of a patient with SCLC with 23 brain metastases that were all treated with SRS alone, without detriment to intracranial control, cognitive impairment, or overall quality of life.

## Case presentation

**Figure 1:**
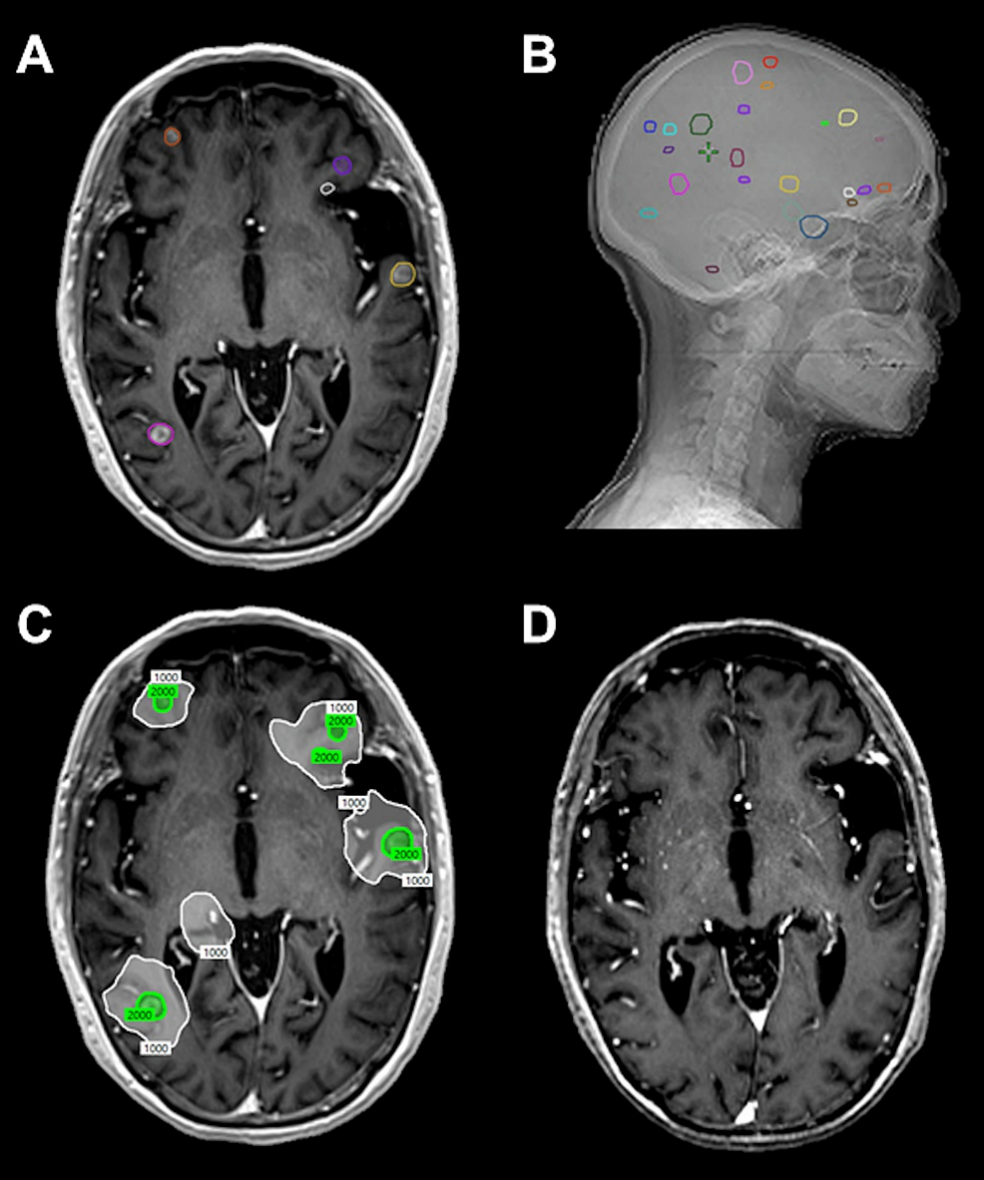
Treatment Planning and Follow-Up Imaging (A) Representative axial slice from treatment planning magnetic resonance imaging showing five treated gross tumor volumes. (B) Representative lateral digitally reconstructed radiograph showing the distribution of 23 treated lesions. (C) Representative axial slice showing radiation treatment plan isodose lines including the prescription dose (20 Gy, green) and the 50% isodose line (10 Gy, white). (D) Representative axial slice from follow-up magnetic resonance imaging showing treatment response and disease surveillance.

A 78-year-old female with an excellent performance status was initially diagnosed with extensive-stage SCLC (stage IVB, cT3, cN3, pM1c). Magnetic resonance imaging (MRI) of the brain at the time of diagnosis was negative for intracranial disease. She initiated treatment with systemic therapy in the form of carboplatin/etoposide/atezolizumab. Additionally, due to significant respiratory symptoms, palliative radiation therapy was given to the left lung to a total dose of 45 Gray in 15 fractions, followed by stereotactic body radiation (SBRT) to the left ischium for pain control. Following the completion of four cycles of carboplatin/etoposide/atezolizumab, she was transitioned to maintenance atezolizumab. Unfortunately, three months later, she presented with mild dizziness and blurry vision, and a brain MRI was obtained. She did not have any additional headaches or new-onset focal neurologic changes. MRI revealed 14 enhancing intracranial lesions, consistent with metastasis, the largest of which measured 13.9 mm. She was therefore referred to radiation oncology for consideration of local therapy. After a discussion with the patient, she declined WBRT in favor of SRS, citing the risk of chronic fatigue, neurocognitive dysfunction, and permanent hair loss as reasons for her decision. At the time of computed tomography (CT) simulation two weeks after her initial MRI, a repeat MRI was obtained, which revealed that the total number of lesions had increased to 23 lesions. Following a discussion with the patient, the decision was made to proceed with the planned course of SRS with the understanding that future WBRT would be indicated if she were to progress further. SRS was planned for a single fraction of 20 Gy to each lesion, using two isocenters, with image guidance in the form of cone-beam CT to maximize setup accuracy and treatment volume targeting. Treatment planning images are shown in Figure 1A-1C. Relevant treatment planning dosimetry is shown in Table 1.

**Table 1:** Single-Fraction SRS Radiation Treatment Plan Dosimetric Metrics SRS, stereotactic radiosurgery; Gy, Gray; VX, volume receiving at least X Gy; D_max_, max dose; D_mean_, mean dose *One lesion was abutting the hippocampus.

Metric	Value	Traditional single-fraction SRS constraint
Brain V10 Gy	125.7 cc	<12 cc
Brain V12 Gy	69.8 cc	<10 cc
Brainstem D_max_	8.66 Gy	<15 Gy
Cochlea D_max_	3.17 Gy	<9 Gy
Optic pathway D_max_	6.06 Gy	<8 Gy
Hippocampus D_max_	20.9 Gy*	Not defined
Hippocampus D_mean_	7.2 Gy	Not defined

Three-month follow-up after treatment, with repeat MRI, demonstrated an overall positive response with treated lesions mostly eradicated (Figure 1D) and no evidence of radiation-induced toxicity including radionecrosis. There were two new lesions found, one located in her right cerebellum and one within the right temporal lobe. She did note continued dizziness and vision changes but continued to have no headache, weakness, or numbness. The identified lesions were subsequently treated with repeat SRS to a dose of 20 Gy in one fraction. Unfortunately, shortly after completing her second course of SRS, she developed extracranial disease progression and ultimately succumbed to her illness nine months after her initial course of SRS.

## Discussion

As with many other malignancies, optimizing treatment to be convenient while minimizing a patient’s adverse effects is something to continuously strive for. Although WBRT is the standard option for treating brain metastases from SCLC, SRS might still achieve comparable or even better results. Having the ability to avoid critical structures in the area including the normal brain, hippocampus, optic chiasm, optic nerves, and brainstem, it is beneficial to seek to expand indications for SRS, including in SCLC.

One of the main concerns about utilizing SRS over WBRT is the lack of prophylactic exposure to areas that had not shown up on imaging. As metastasis may be present prior to being visible on a scan, it is imperative to consider the potential for widespread intracranial involvement. This risk theoretically increases with the number of metastases, as it might represent a surrogate for the overall extent of intracranial disease, both macroscopic and microscopic. However, recent literature in support of SRS compared to WBRT calls into question whether this potential for increased intracranial control is worth the added toxicity if a patient can be closely monitored with serial MRI and undergo additional salvage radiation if needed [10,11]. However, the use of SRS for more than 15 lesions, particularly with SCLC histology, rightly remains highly controversial. Many factors might go into the decision to offer SRS, including patient perspective, performance status, the overall burden of disease, and response to systemic therapy. These features should be considered in future studies that seek to identify candidates for SRS in lieu of WBRT.

In addition to concerns for disease control, treatment of numerous lesions with SRS raises concerns for the ability to meet dosimetric constraints related to toxicity. As can be seen in Table 1, all dosimetric constraints were satisfied, except for integral brain dose, which is not overly surprising given the total number of lesions treated. Importantly, the provided constraints were developed by Minniti et al. [13] to limit the risk of radionecrosis. However, this analysis was comprised only of patients with 1-3 metastases, with 61% having a single metastasis. It is plausible that the actual predictive constraints might scale with the total number of lesions, given that the dose is spread out more throughout the brain, and therefore, it has been our institution’s practice to not use those constraints as hard stops. When using an alpha/beta ratio of two, the 10 Gy threshold computes to a biologically equivalent dose (BED) of 60 Gy, while whole-brain radiation (30 Gy/10 fractions) results in a BED of 75 Gy, and thus, the dose-toxicity relationship is likely more nuanced than what published constraints currently represent.

Fortunately, in the presented case, despite treating a total of 25 lesions over two courses of SRS, intracranial disease progression did not represent a detriment in the patient’s overall treatment course, nor did she experience radionecrosis. Indeed, at the time of her death, her intracranial disease was controlled, with extracranial disease representing her life-limiting complication. This case demonstrates that SRS might still produce favorable results even in patients with SCLC and more than 10 brain metastases. There continues to be a need for further evaluation to determine the extent of disease that can be safely and effectively treated with SRS and when it should be utilized. This question will start to be addressed with the randomized ENCEPHALON Trial, which compares WBRT alone versus radiosurgery for SCLC patients with 1-10 brain metastases [14].

## Conclusions

As the ongoing debate continues within radiation oncology regarding the efficacy and use of SRS versus WBRT, this is the first report that may bring to light the consideration for SRS in SCLC patients with multiple brain metastases. As future studies continue to address the question of each treatment’s utilization, specifically for SCLC, this case may provide some guidance in determining select cases who may be good candidates for SRS, when presenting with multiple brain metastases in the setting of SCLC.
